# Mechanical Evaluation of Two Hybrid Locking Plate Designs for Canine Pancarpal Arthrodesis

**DOI:** 10.1155/2021/2526879

**Published:** 2021-09-02

**Authors:** Ivan Zderic, Peter Varga, Ursula Styger, Ludmil Drenchev, Boyko Gueorguiev, Erik Asimus, W. Brian Saunders, Michael Kowaleski, Randy J. Boudrieau, Loic M. Dejardin

**Affiliations:** ^1^AO Research Institute Davos, Clavadelerstrasse 8, 7270 Davos Platz, Switzerland; ^2^Institute of Metal Science, Equipment and Technologies, Bulgarian Academy of Sciences, Shipchenski Prohod Str., 67, 1574 Sofia, Bulgaria; ^3^Ecole Nationale Vétérinaire de Toulouse, 23 Chemin des Capelles, 31300 Toulouse, France; ^4^College of Veterinary Medicine & Biomedical Sciences, Texas A&M University, 4474 TAMU, College Station, TX 77843, USA; ^5^Cummings School of Veterinary Medicine, Tufts University, 200 Westboro Rd., North Grafton, MA 01536, USA; ^6^College of Veterinary Medicine, Michigan State University, 736 Wilson Rd., East Lansing, MI 48824, USA

## Abstract

Hybrid locking pancarpal arthrodesis plates were designed with either a round (RH) or an oval (OH) radiocarpal hole, the latter allowing varied screw positioning. Due to concerns about potential decreased structural properties of the OH design, our aim was to compare the mechanical behavior of the contrasting plates using combined finite element analysis (FEA) and mechanical testing. Pancarpal arthrodesis plates with RH or OH design were assigned to three fixation techniques (*n* = 6), prebent at 20°, and fixed to canine forelimb models with simulated radius and radiocarpal and 3rd metacarpal bones. OH plates were instrumented with a radiocarpal screw inserted either most proximal (OH-P) or most distal (OH-D). Specimens were axially loaded to 300 N over 10 ramped cycles at 0.5 Hz. Plate strains were measured with strain gauges placed at areas of highest deformations as predicted by FEA under identical loading conditions. FEA predicted the highest strains (*μ*m/m) adjacent to the radiocarpal hole (2,500 [RH], 2,900 [OH-P/OH-D]) and plate bending point (2,250 [RH], 1,900 [OH-P/OH-D]). Experimentally, peak radiocarpal hole strains were not influenced by the OH screw position (3,329 ± 443 [OH-P], 3,222 ± 467 [OH-D]; *P* = 0.550) but were significantly higher compared to the RH design (2,123 ± 154; *P* < 0.001). Peak strains at the bending point were significantly lower for OH-P (1,792 ± 174) and OH-D (1,806 ± 194) versus RH configurations (2,158 ± 114) (*P* ≤ 0.006). OH plates demonstrated highest peak strains next to the radiocarpal hole and were associated with more heterogenous plate strain distribution. Structural weakening associated with radiocarpal OH plate design could result in decreased fixation strength and increased risk of plate fatigue failure.

## 1. Introduction

Pancarpal arthrodesis is a well-established procedure used for treatment of a variety of canine carpal disorders including hyperextension injuries, severe fractures or luxations, end-stage osteoarthritis, and selected neurological deficits [1–5]. Although numerous internal and external stabilization techniques have been described, the most common procedure relies on a dorsally applied plate [6, 7]. Despite a biomechanical disadvantage inherent to plate placement across the compression side of the joint, this location is often preferred over the palmar positioning due to the presence of palmar neurovascular and tendinous structures encountered during palmar plating [1, 8]. Dorsally applied plates also have biological disadvantages due to their large size, which may result in increased soft-tissue tension during wound closure. To limit the risk of plate yield or fatigue failure due to cyclic bending, carpal fusion at a straight angle of 0° extension has been recommended [6]. Yet, from a physiological standpoint, radiocarpal joint fusion at approximately 15° to 20° of extension is preferred as it optimizes paw placement during stance and limits flexor tendon pain [9–11]. Regardless of the arthrodesis angle, a dorsally positioned plate is at a mechanical disadvantage that increases plate stress, which can contribute to failure at the level of the radiocarpal joint.

**Figure 1:**
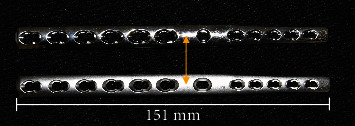
Limited contact locking pancarpal arthrodesis plate 2.7/3.5 mm with round (top) and oval (bottom) radiocarpal hole, 12 holes, and length 151 mm. Double arrow indicates the bending point for each plate.

To address the limitations of most current plating methods, new designs have been developed, including a hybrid plate with a distally tapered profile for both its width and thickness. This plate, featuring a round radiocarpal hole, provides for larger screws to be placed in the radius and smaller screws in the 3^rd^ metacarpal, thus optimizing stability while limiting the risk of metacarpal fractures and wound dehiscence [12–14]. Recently, two new pancarpal arthrodesis plates were developed (DePuy Synthes, Paoli, PA) to take advantage of this tapered profile and, in addition, locking screw technology. Due to the angle-stable plate-screw interface, locking plates act as single beam constructs, which have been shown to be stronger than size-matched load-sharing beam constructs such as compression plates [15]. Furthermore, the risk of construct failure secondary to standard screw loosening, as observed with compression plating, is reduced with locking plate constructs. Screw stability with a locking construct does not rely on frictional forces between the plate and bone but on direct engagement of the screw with the plate hole [16]. The initial novel plate was designed with a round radiocarpal hole. The subsequent version featured an oval radiocarpal hole to allow for improved screw placement into the radiocarpal bone with plate position adjustments (Figure 1). Whereas the latter plate design may facilitate plate application, it could diminish its mechanical strength by lowering the area moment of inertia near the point of maximum stress. While the mechanical superiority of hybrid compression plates over standard limited contact compression plates has been demonstrated in a recent study [17], comparisons between the two novel designs are lacking. Furthermore, the effect of an oval radiocarpal hole on the structural parameters of the plate has not been tested.

Therefore, the aim of this combined in silico and experimental study was to compare the mechanical behavior of hybrid locking pancarpal arthrodesis plates with oval (OH) versus standard round (RH) radiocarpal holes under the hypotheses that plate strain, construct compliance, and angular deformation under loading would be higher (1) for OH versus RH radiocarpal hole designs and (2) for the proximal (OH-P) versus distal (OH-D) screw locations within the OH design.

## 2. Materials and Methods

### 2.1. Specimen Preparation

**Figure 2:**
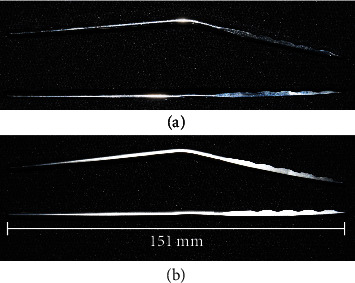
Lateral view of the limited contact locking pancarpal arthrodesis plate 2.7/3.5 mm with round (a) and oval (b) radiocarpal holes in its prebent (top) and original straight (bottom) shapes.

Six RH and OH hybrid locking pancarpal arthrodesis plates accepting 3.5 mm and 2.7 mm screws were manufactured from implant-grade stainless steel (316L) by DePuy Synthes (Paoli, PA). The implants were consistently prebent for 20° joint fusion angle using a custom-made bending press (Figure 2) [17]. The press was designed to consistently and accurately bend the plates at a point centered at the solid plate section between the distal radial and radiocarpal holes (Figure 1). All bent plates were fixed to solid (25 mm diameter) cylindrical phenolic-cotton fabric composite bone model sections (Canevasite HGW 2082, Amsler & Frey AG, Schinznach-Dorf, Switzerland) simulating the radius and radiocarpal and 3^rd^ metacarpal bones of a midsize 30 kg dog. The lengths of the bone model substitutes measured 171 mm, 10 mm, and 123 mm, respectively. Each plate was fixed to the radius with 3.5 mm locking screws in plate holes 1 (proximal), 3, 5, and 6, whereas the metacarpus was fixed with 2.7 mm locking screws in holes 1 (proximal), 2, and 5. Finally, the radiocarpal bone was fixed to the plate with a 3.5 mm cortical screw through the radiocarpal hole. Pilot holes of 2.8, 2.0, and 2.5 mm were predrilled in the bone models prior to insertion of the respective screws. All 3.5 mm screws (locking and cortex) were tightened at a final torque of 3 Nm, whereas the 2.7 mm locking screws were locked at 1.5 Nm. The plates with OH were investigated with one of two configurations of the radiocarpal screw fixed in either the most proximal (OH-P) or most distal (OH-D) hole position. To ensure homogeneous initial conditions, three constructs were investigated with OH-P configuration and three with OH-D configuration prior to the final testing with these respective configurations.

### 2.2. Finite Element Analyses

A priori finite element analyses (FEA) were conducted in Abaqus 6.13 (SIMULIA, Dassault Systèmes SE, Velizy-Villacoublay, France) to identify areas with the highest strains, i.e., the most probable failure locations of the plates. To mimic the subsequent experiments, the simulations were split into two parts, corresponding to (1) plate bending in the custom press and (2) axial bending of the instrumented constructs during loading.

**Figure 3:**
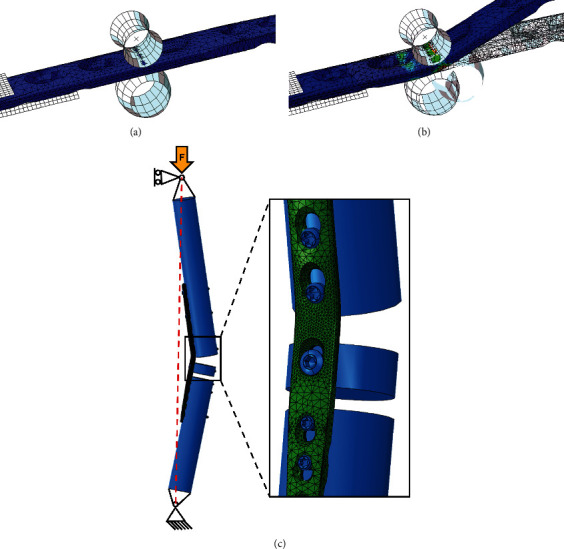
Illustration of the two-step FEA with virtual prebending of the oval hole (OH) plate and simulation of in vivo loading conditions by axial compression; OH plate fixed between two rollers before (a) and after (b) the bending process. Residual stresses derived from the prebending phase were transferred for the second step of the FEA. (c) Boundary conditions for simulation of in vivo loading conditions were based on the experimental set-up. Magnification shows radiocarpal hole and bending point with applied refined mesh (0.8 mm) in this region.

The first simulation included the plate and two rolls of the press modelled as rigid bodies (Figure 3(a)). The original unbent implant geometry was defined based on computer-aided design (CAD) files provided by the manufacturer, where the threads were removed from the locking holes. The models were meshed with quadratic tetrahedral elements of 2 mm edge length, refined to 0.8 mm around the areas of interest, being the planned bending zone, the region of the radiocarpal hole, and all other holes occupied with screws. The created mesh sizes relied on a previously performed biomechanical study [18], where a convergence test for accurate strain prediction was conducted. Consequently, repeating a convergence test was not performed, particularly because the FEA was used for a qualitative assessment of the areas associated with highest strains. An elastic-perfect plastic material model was used with Young's modulus of 210 GPa, Poisson's ratio of 0.3, and yield stress of 1100 MPa [19, 20]. Frictionless contact was defined between the rolls of the press and the implant surfaces. The bending process, targeting a 20° extension fusion angle, was simulated by constraining the motion at the proximal side of the plate and mimicking the displacements of the moving roll of the custom press (Figure 3(b)), with subsequent release of the constraints to obtain the final shape including the plastic deformations only. Geometrical nonlinearities were considered.

The second simulation was aimed at predicting the deformations around the regions of interest by reproducing the experimental set-up of construct testing (Figure 3(c)). The bent plate models were imported from the first simulation, including the residual stresses, as a predefined field input. The plates were virtually instrumented on cylindrical bone models mimicking the bone substitutes using the same number and location of screws to be used experimentally. The bone replicas were modelled with an elastic modulus of 8 GPa [21]. The simplified screw models without threads were linked to the bone models and plate holes via bonding constraints. The compression screw at the radiocarpal hole was simulated using contact conditions between its screw head and plate hole with a friction coefficient of 0.2 and a load of 500 N simulating compression [18]. In accordance with the experiments, the OH plate was investigated both with OH-P and in OH-D configurations. The models were analyzed for peak plate strains in the loaded condition around the radiocarpal hole region and at the bending site.

### 2.3. Experimental Quasistatic Testing

Linear strain gauges (LY81-0.6/120, HBM, Darmstadt, Germany) were fastened in the radiocarpal region of the plates at areas associated with highest deformations, as predicted by the FEA, namely, at the bending point and next to the radiocarpal hole. Two strain gauges were attached next to the radiocarpal hole of the OH plates, one at the hole site occupied by the screw and the other at the opposite unoccupied site of the hole.

**Figure 4:**
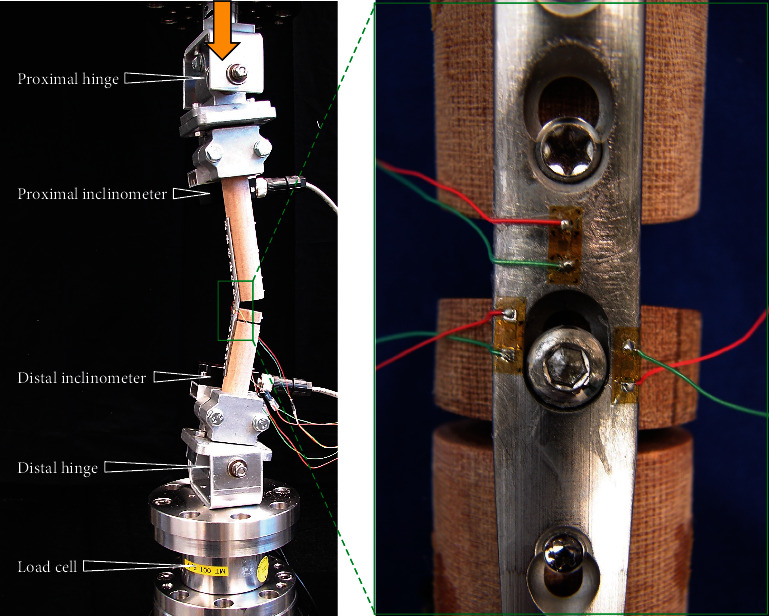
Test set-up with a specimen mounted for mechanical testing. Vertical arrow indicates loading direction. Magnification shows radiocarpal screw placed in the distal aspect of the oval plate hole (OH-D) construct with applied strain gauges at the weakest locations predicted by FEA: the plate bending point, adjacent to occupied/unoccupied radiocarpal hole region.

Mechanical testing of the specimens was performed using a servohydraulic material testing machine (Mini Bionix 858, MTS Systems, Eden Prairie, MN) equipped with a 4 kN load cell (Figure 4) Articulated cups with rotational axes parallel to each other were used proximally and distally to allow free specimen rotation in the sagittal plane. Each cup was equipped with an inclinometer (8.IS40.23321, Kübler, Villingen-Schwenningen, Germany). The distances between the bending point and the proximal and distal articulations of the cups were identical for all specimens and guaranteed consistent lengths of the proximal and distal lever arms with respect to the radiocarpal bone. To reproduce postoperative activity during trotting of a 30 kg dog, the specimens were loaded under axial bending along the machine axis to 300 N compression over 10 cycles at 0.5 Hz [22, 23].

Axial displacement and load were recorded from the MTS transducers together with inclinometer and strain gauge output data at 128 Hz. The last cycle of each test was considered for data evaluation. Based on MTS and inclinometer data, construct compliance (CC [°/N]) and maximum angular deformation (AD [°]) were computed. Strain gauge outputs were used to estimate the peak plate strains (PPS [*μ*m/m]).

### 2.4. Statistical Analysis

Statistical analysis was performed using SPSS software (version 23, IBM SPSS, Armonk, NY). Mean and standard deviation (SD) were calculated for each parameter of interest.

The Shapiro-Wilk test was conducted to ascertain a normal data distribution within all fixation techniques. One-Way Analysis of Variance (ANOVA) with Bonferroni post hoc test was conducted to identify significant differences between OH and RH plates, whereas the differences between OH-P and OH-D were screened with Paired-Sample *t*-test. A level of significance was set to 0.05 for all statistical tests.

## 3. Results

**Figure 5:**
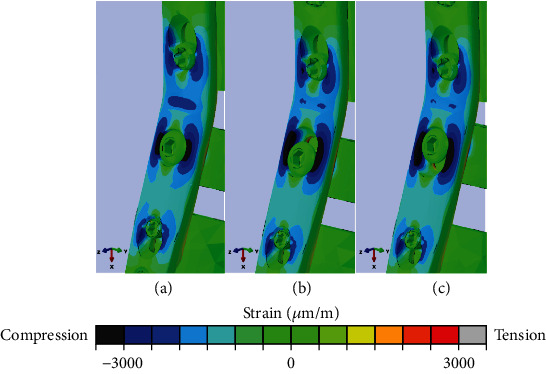
Principal strains with color-coded strain scale (bottom) in the radiocarpal hole region and at the bending point under 300 N axial compression, shown for round hole (RH) (a), screw insertion at distal margin of oval hole (OH-D) (b), and screw insertion at proximal margin of oval hole (OH-P) (c), indicating for RH a lower strain magnitude next to the radiocarpal hole and a higher strain magnitude at the bending point, compared to both OH-D and OH-P.

The FEA predicted the highest strains next to the radiocarpal hole (2500 *μ*m/m [RH], 2900 *μ*m/m [both OH-P/OH-D]) and at the bending point (2250 *μ*m/m [RH], 1900 *μ*m/m [OH-P/OH-D]) (Figure 5). While the RH plate was associated with higher strains at the bending point, lower strains were predicted next to the RH compared to both OH-P and OH-D configurations. For the latter two, no significant difference in plate strains was detected.

**Figure 6:**
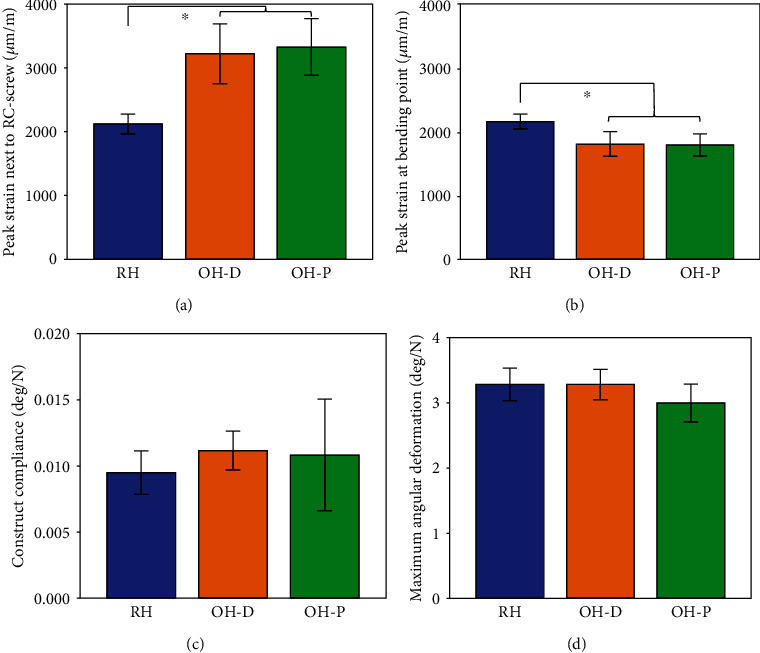
Outcome measures for the four parameters of interest derived from experimental testing, namely, peak strain next to RC hole (a) and at bending point (b), as well as construct compliance (c) and maximum angular deformation (d), shown in terms of mean and SD values for each plate and screw configuration separately. ∗ indicates significant differences between fixation techniques.

Experimentally, mean peak radiocarpal hole strains in the OH plates were not significantly influenced by the radiocarpal screw position (3329 ± 443 *μ*m/m [OH-P], 3222 ± 467 *μ*m/m [OH-D], *P* = 0.550) but were significantly higher for both these screw configurations compared to RH plates (2123 ± 154 *μ*m/m, *P* < 0.001). In contrast, peak strains at the bending point were significantly lower (*P* ≤ 0.006) for OH-P (1792 ± 174 *μ*m/m) and OH-D (1806 ± 194 *μ*m/m) compared with RH (2158 ± 114 *μ*m/m). Construct compliance and maximum angular deformation demonstrated no significant differences between the three constructs (*P* ≥ 0.123), (Figure 6).

The predicted strains by FE analyses deviated from the experimental mean values measured at the bending point by 4% for RH, 6% for OH-D, and 5% for OH-P. At the radiocarpal hole, these deviations amounted to 18% for RH, 13% for OH-D, and 10% for OH-P.

## 4. Discussion

This study evaluated the biomechanical performance of two hybrid locking plate designs for canine pancarpal arthrodesis with either a round or an oval radiocarpal hole by means of combined FEA and experimental quasistatic axial loading. In addition, the effect of occupying the two extreme ends of the oval radiocarpal hole by a screw was investigated. As hypothesized, the RH plates demonstrated significantly lower peak strains next to the radiocarpal hole compared with OH plate configurations, the latter demonstrating overall highest peak strains at that specific region. The lower biomechanical performance of the OH plates is a logical consequence of their reduced cross-sectional area and, therefore, smaller area moment of inertia around the radiocarpal hole region. Although no plastic deformation was detected during testing, the OH plates were associated with strains higher than 0.3% (3000 *μ*m/m) which was 50% more than the magnitudes of approximately 0.2% (2000 *μ*m/m) detected in RH plates. This infers that under the same loading conditions, internal stresses in OH plates are higher than those in RH plates. Under conditions of loading during the convalescence period, these higher stresses would likely render OH plates more susceptible to fatigue failure compared to RH plates.

Plate fixation is generally recommended to stabilize the arthrodesis construct [4]. Since there is evidence that plate breakages after pancarpal arthrodesis can be minimized by fixing the plate to the radiocarpal bone with a screw [4], this supplemental fixation has become the standard-of-care. Pancarpal arthrodesis plates feature a radiocarpal screw hole allowing fixation to this bone, which has been shown to increase compression across all of the joints of the carpus [24]. However, given the fact that the carpus is a complex structure composed of many small bones, proper screw placement may not be ensured when using a round radiocarpal hole. For that purpose, a novel plate, characterized with an oval radiocarpal hole, was designed to give the surgeon more flexibility for plate fixation in this region. This hole allows a 2.5 mm screw shift in the proximodistal direction and enables additional screw angulation of up to 25°. This freedom of screw placement facilitates optimal purchase in the radiocarpal bone without significantly changing plate strains—neither FEA nor experimental testing revealed an effect of moving the radiocarpal screw from proximal to distal. Nevertheless, the radiocarpal hole is one of the most susceptible regions of plate failure because of its location near the apex of the plate bend, which also results in high induced bending moments and some structural weakening of the plate during bending. This has been substantiated by previous clinical studies reporting plate breakage after pancarpal arthrodesis [4, 6, 7, 9, 24, 25]. An option to mitigate plate strains is to use external coaptation with a cast, although its clinical relevance remains debatable due to adverse side effects, such as soft-tissue injuries and increased treatment costs [26, 27]. We anticipate that a design iteration may better alleviate the adverse effects observed with the OH plate design while maintaining its mechanical strength. For example, the shape of the plate could be modified in this region by enlarging the side flanks, making them thicker, without compromising the sliding oval hole.

Notwithstanding the aforementioned mechanical shortcoming of the OH plate design, it should be noted that plate breakages are less frequently reported than other implant-related complications, such as screw loosening or breakage [4, 7, 12] or postoperative fractures of the third metacarpal bone, the latter found to correlate with length of plate coverage [13]. In their retrospective cohort study comparing the outcomes of pancarpal arthrodesis performed in 219 dogs, using either hybrid compression plate or a variation, Bristow et al. [2] found plate breakage to occur in less than 1% of the cases.

Considering that screw loosening represents a major problem associated with pancarpal arthrodesis [3, 24, 28], both plate types (OH and RH) provide advantages by enabling locking of all screws, except for the radiocarpal hole which requires use of a standard cortex screw. The expected advantages of the locking plate design are enhanced resistance against screw loosening and/or pullout combined with reduced contact area between the plate and the bone, which allows for better blood supply preservation dependent upon the method of plate application [29, 30]. However, it should be noted that locking plates were originally developed for fracture fixation in osteoporotic bone to enhance stability. In view of this, the overall construct stability in normal bone may not be increased compared to compression plating [30, 31].

Since nondestructive testing was conducted, the present study indicates that overall construct strength measurements could be misleading and highlight the importance of local assessment of the mechanical behavior of specific bone-implant construct areas. Although construct compliance and maximum angular deformation revealed no significant differences between fixation techniques, it was evident that when using the same overall plate design, the mechanical performance of specific implant regions under loading was different. It can be reasonably assumed that minor design iterations could mitigate these changes. Local strain measurements are therefore appropriate when used for prediction of ultimate materials strength. In addition, we propose that a preliminary FEA is helpful in accurately allocating strain gauge application to regions associated with elevated surface strains.

This study best compares to the one performed by Guillou et al. [17]. In their biomechanical study, the authors compared a limited contact compression plate to a hybrid pancarpal arthrodesis compression plate in a cadaveric model using a set-up, loading protocol, and method of strain measurement which served as the template for the present study design. The strains in their study were measured at the bending location of the plates. Mean strain values in the two groups under 300 N compression loading were 1098 *μ*m/m and 1736 *μ*m/m, which is somewhat smaller than the results of the present study. This could be explained by the potentially higher moment of inertia and bending stiffness, i.e., higher plate thickness inherent with the compression plates used in the cited study.

Some limitations of the current study need to be disclosed. First, due to technical restrictions, strain gauge measurements were not concomitant with the bending process. However, these residual strains were simulated with FEA. Therefore, only a proportional comparison between the two approaches was feasible. It remains open as to which amount residual plate stresses and strains are generated during bending. Second, plate strains were measured experimentally only at the bending point and next to the radiocarpal hole in the non-weight-bearing carpal region. The more peripheral weight-bearing areas—the fourth radial and first metacarpal screw holes—were neglected, although some elevated strains were indicated by FEA. Similarly, the more common failure modes of screw loosening or breakage, as well as metacarpal fracturing were not investigated in this study, which focused on the potential compromise of the structural strength around the radiocarpal hole. Third, strain gauges were applied manually to the compression side of the plates. While this could have led to variations regarding their positioning relative to the plates, data was associated with relatively low scatter. Furthermore, since tension is the loading mode where ductile materials, such as stainless steel, are prone to fail first [32], measuring plate strains on the tension side might have provided more conclusive data on the risk of plate failure under the current loading protocol. However, the compression side of the plate was chosen based upon the restricted space on the tension side, which was covered by the bone models. Forth, a highly simplified forelimb model was considered in silico and experimentally, using artificial bone substitutes with no load-sharing between the single bones in the metacarpal region. Although the implementation of load-sharing between the three fragments would have represented more accurately the clinical scenario, it may have led to less pronounced differences in plate strains among the different plate designs and screw configurations, reducing the power of the statistical analyses. Therefore, it was found beneficial to subject the specimens to such a worst-case scenario. In addition, although the strength of the solid bone models has overestimated real bone characteristics, their usage was deemed appropriate as it allowed focusing on the effect of plate design iterations by reducing adverse variables to a minimum. Fifth, the FEA used assumptions of the screw-plate locking principles and the screw threads. Finally, a true comparison of the biomechanical performance of the two plate designs in terms of failure prediction is only feasible under cyclic loading to failure, which remains to be investigated.

One of the main findings of the present study is the appropriateness of plate strain prediction by FEA. The discrepancy between FEA prediction and quantitative experimental data was as low as 3% and no greater than 18%. This suggests that our FE model can be considered as suitable for allocating areas with increased strains. Furthermore, the low plate strain scatter data demonstrated accurate and repeatable strain gauge application.

Further studies are required to investigate the effect of plate design iterations on fatigue failure risk. Should that risk remain similar between plates, the radiocarpal oval hole design could provide surgeons with more options regarding plate/screw positioning in dogs of various size. Conversely, design modifications around the oval radiocarpal hole would be necessary to remedy focal weakening of the plate structural properties if confirmed. Finally, clinical studies investigating the clinical performance of both plates would be highly beneficial to confirm the validity of the present study.

## 5. Conclusions

From a biomechanical perspective, oval hole locking pancarpal arthrodesis plates demonstrated the overall highest peak strains next to the radiocarpal hole and were associated with more heterogenous plate strain distribution between the radiocarpal hole and bending point areas compared to round hole plates. The slight structural weakening due to radiocarpal OH plate design led to increased deformation at that location. From a clinical perspective, radiocarpal OH plate design could result in decreased fixation strength and increased risk of plate fatigue failure.

## Data Availability

Data are available on request through the corresponding author.
